# Temperature-modulated persistent dsRNA phage infection selectively excludes superinfection and promotes generation of new viral variants

**DOI:** 10.1128/spectrum.04029-25

**Published:** 2026-05-20

**Authors:** Meri M. Salomaa, Sally K. Chesnut, Xiaoyu Sun, Sari Mäntynen, Minna M. Poranen

**Affiliations:** 1 Department of Molecular and Integrative Biosciences, Faculty of Biological and Environmental Sciences, University of Helsinki 3835, Helsinki, Finland; University of California San Diego, La Jolla, California, USA

**Keywords:** double-stranded RNA virus, cystovirus, bacteriophage phi6, persistence, carrier cell, carrier state, superinfection exclusion, reassortment, reverse genetic analysis

## Abstract

Persistent viral infections are common in nature and can play a significant role in the ecology and evolution of the host organism. However, our understanding of these prolonged virus-host interactions remains limited. This study characterizes the persistent infection strategy of Pseudomonas phage phi6, an enveloped virus with a tri-segmented, double-stranded (ds)RNA genome that infects *Pseudomonas syringae*. Phi6 is predominantly lytic but can alternatively establish a non-productive chronic infection (carrier cell state) in which viral particles assemble and replicate viral RNA without causing host cell lysis and viral progeny release. We utilized reverse genetics tools to produce stable phi6 carrier cells and identified genetic factors essential for carrier cell formation. We found that the persistent phi6 infection does not significantly impact host growth at the optimal growth temperature. Nevertheless, growth temperature may regulate the switch between the carrier cell state and lytic infection. In addition, the persistent phi6 infection protects the host bacterium against secondary phi6 infections, providing an apparent fitness benefit for the host. Gene knockout assays suggested that phi6 gene *8,* encoding the viral major outer capsid protein P8, contributes to the superinfection exclusion effect. However, the persistently infected bacterial cells remained susceptible to other dsRNA phages, enabling genetic exchange between related phages. Accordingly, P8 of phi6 specifically inhibited transcription by phi6 nucleocapsids but did not suppress transcription of a related phage in an *in vitro* transcription assay. This study sheds light on the underlying molecular mechanisms and potential ecological implications of persistent dsRNA phage infection in plant pathogenic *Pseudomonas*.

**IMPORTANCE:**

Double-stranded (ds)RNA viruses infect a wide range of hosts, including bacteria, fungi, plants, and animals, and this group includes many important pathogens. While acute infections of these viruses are well understood, less is known about dsRNA virus persistence. We studied persistent infection of a bacterial dsRNA virus, cystovirus phi6, in *Pseudomonas syringae*. In this virus-host interaction, intracellular phage particles are continuously produced without host cell lysis, deviating from other known persistent phage infection strategies. Our findings show that persistent dsRNA virus infection can benefit the host: persistence does not affect host growth but protects it from subsequent phi6 infections. Nevertheless, the persistently infected host remains susceptible to other viruses, promoting the formation of new viral genotypes, and highlighting the importance of persistent infections in viral evolution. Furthermore, understanding the carrier cell phenomenon is relevant for the development of phi6-based biocontrol of *Pseudomonas* infections in crops.

## INTRODUCTION

Acute viral infections cause many devastating diseases in humans, animals, and economically important crops, and the associated virus-host interactions have been extensively studied. However, in addition to acute productive infection, resulting in the release of infectious progeny, viruses can establish persistent interactions with their host. This has been well documented for DNA bacteriophages (lysogeny, pseudolysogeny), as well as for many eukaryotic DNA viruses (latent infection), and the ecological and evolutionary role and medical impact of such virus-host interactions have been recognized (1–3). Nevertheless, persistence is likely a common infection strategy also among dsRNA viruses, which employ intra-capsid genome replication and transcription strategies. An extreme example is fungal dsRNA viruses that persistently infect their host without having an alternative productive infection cycle. Moreover, persistent infection mode has been described for several animal and plant dsRNA viruses (4–11), as well as for dsRNA bacteriophages of the *Cystoviridae* family (12–15).

Persistent interactions between DNA phages and their bacterial hosts have been shown to manifest in various forms. In contrast to virulent (strictly lytic) phages, temperate phages can also enter a lysogenic cycle, in which the phage genome is either integrated into the host chromosome or is in a plasmid-like state (16–18). In the lysogenic cycle, the phage genome replicates passively as the host cell divides, allowing the phage to persist within host populations over extended periods. Evidence has also emerged on alternative persistent phage lifestyles that deviate from the canonical lytic-lysogenic binary. For instance, pseudolysogeny is a stalled phage development stage, reported for several dsDNA phages, in which the unintegrated phage genome is asymmetrically passed on to one of the daughter cells during cell division (2, 19–21). Furthermore, filamentous ssDNA and pleomorphic dsDNA phages establish a productive chronic infection in which progeny phage particles are released through the host cell membrane without lysing the cell (22–26). In addition, prolonged host interactions have been described for several dsRNA phages of the *Cystoviridae* family (12, 13, 15), and for ssRNA phage LeviOr01 (27), suggesting that persistence could be a frequent phenomenon not only in DNA phages, but also in RNA phage populations. Nevertheless, the mechanistic basis and eco-evolutionary implications of prolonged infection strategies of RNA viruses have remained largely uncharacterized.

Cystoviruses are enveloped viruses having a trisegmented dsRNA genome (S, M, and L) and a double-layered protein capsid, called the nucleocapsid (NC) (28, 29) (Fig. S1). These viruses infect *Pseudomonas* bacteria, mainly plant pathogenic *P. syringae* strains that are responsible for many agriculturally important crop diseases, but some isolates also infect *P. aeruginosa* (28). During infection of the *Pseudomonas* host cell, the cystoviral dsRNA genome is replicated and transcribed within a protein capsid, called the polymerase complex, which forms the innermost layer of the virion. Instead of having a strictly lytic lifestyle (Fig. S2A), some cystoviruses can also establish a persistent non-productive chronic infection, known as a carrier cell (19) (Fig. S2B). In carrier cell infection, polymerase complexes form, replicate, and transcribe the viral genome, and stably remain within the host cell without cell lysis (Fig. S2B). Thus, in the persistent cystoviral infection, the viral dsRNA genome is continuously expressed and replicated, and new viral particles are formed, which deviates largely from the persistent interactions between DNA phages and their bacterial hosts. Cultures of cystovirus carrier cells form a carrier state in which the virus and host are continuously propagated (19). In the carrier state culture, the lytic life cycle may be spontaneously activated in individual carrier cells, resulting in the release of progeny phages into the culture medium. Initially, spontaneous cystoviral carrier state cultures were reported for Pseudomonas phage phi6 in its *P. syringae* pv. *phaseolicola* HB10Y host (13, 15), and have recently been described for dsRNA phage phiNY of *Microvirgula aerodenitrificans* (12). Persistent cystovirus-host interaction can also be established by incorporating a reporter gene into the viral genome (30, 31) or by introducing mutations into the gene encoding the viral lytic enzyme (32). The defective lytic enzyme prevents the phage from entering the lytic cycle, facilitating the analysis of coexistence.

The lytic power of cystoviruses could potentially be harnessed to control *P. syringae* infections in crop plants (33). However, the application of cystoviruses as biocontrol agents in agriculture requires an understanding of their alternative non-lytic infection modes. The eco-evolutionary role of dsRNA phage persistence, how it affects the host, virus-host population dynamics, and potentially virus evolution, and the environmental factors that favor persistence also warrant further studies.

## RESULTS

### Generation of spontaneous and synthetic phi6 carrier cell lines

Spontaneous phi6 carrier cell lines were obtained by subjecting *P. syringae* HB10Y cells to an excess of phage. Of the 22 resulting phi6-resistant bacterial strains, four released infectious phages when cultivated in liquid medium overnight (15, Fig. S3). This indicates establishment of carrier cell interaction between phi6 and HB10Y host, and spontaneous activation of the lytic productive cycle of phi6 in a subfraction of the cells during overnight incubation. The presence of the phi6 genome within these HB10Y cells was confirmed by reverse transcription-polymerase chain reaction (RT-PCR) (Fig. S4A; Table S1). These spontaneous carrier cell lines were designated HB10Y(1.2), HB10Y(1.5), HB10Y(2.1), and HB10Y(2.4).

Synthetic phi6 carrier cells were produced by using reverse genetic technique established for phi6 and related cystoviruses (14, 30–32, 34, 35). *P. syringae* LM2691 cells were transformed with plasmids containing complementary (c)DNA copies of the phi6 genome segment L, M, or S, or their derivatives (Table 1). These plasmids do not replicate in *Pseudomonas*, but their transformation results in transient transcription of the viral cDNA sequences by T7 polymerase constitutively expressed in LM2691 (31). The produced viral single-stranded (ss)RNAs have a dual role: they function as messenger (m)RNAs directing viral protein production and as genomic precursor molecules to be encapsidated and replicated by the polymerase complexes (inner capsid) produced in the cell from the L-segment-specific mRNA. Thus, eventually viral replication and reproduction are established, and the virus is maintained in the daughter cells without the presence of the viral cDNA.

**TABLE 1:** Plasmids used in the study

Plasmid name	dsRNA construct (size)	Description^*a*^	Reference
pLM687	phi6 L (6,374 bp)	Wild-type (wt) L-segment encoding proteins P1, P2, P4, and P7 forming the viral polymerase complex	(36)
pLM991	phi6 L_kan_ (7,599 bp)	Kanamycin resistance gene (*nptII*) inserted in the non-coding region of the phi6 L-segment; enables antibiotic selection of the cells carrying the virus	(31)
pLM656	phi6 M (4,063 bp)	wt M-segment encoding envelope-associated proteins P3, P6, P10, and 13	(37)
pLM779	phi6 M_kan_ (5,166 bp)	Kanamycin resistance gene (*nptII*) inserted in the non-coding region of the phi6 M-segment; ; enables antibiotic selection of the cells carrying the virus	(38)
pMS2-9 rep-MP	phi6 M_TMV_ (4,223 bp)	M-segment knockout construct; coding region of the M-segment replaced by sequences derived from tobacco mosaic virus (TMV)	(32)
pLM659	phi6 S (2,948 bp)	wt S-segment encoding major outer capsid protein P8, major envelope protein P9, non-structural protein P12, and lytic enzyme P5	(39)
pMH4	phi6 S_lys_ (2,948 bp)	phi6 S-segment with a defective lytic enzyme gene	(40)
pLM836	phi6 S_kan_ (4,017 bp)	Kanamycin resistance gene (*nptII*) inserted in the non-coding region of the phi6 S-segment; ; enables antibiotic selection of the cells carrying the virus	(31)
pLD18-5 rep-MP	phi6 S_TMV_ (3,268 bp)	S-segment knockout construct; coding region of the S-segment replaced by sequences derived from TMV	(32)
pMH7^*b*^	phi6 S_Δ(5,9,12)_ (2,433 bp)	Gene *8* expressed from the phi6 S-segment	(40)

^*a*^ Phi6-specific sequences are inserted in expression vector pT7T319U under T7 polymerase promoter.

^*b*^ Deletion of genes *5* and *12* in this construct covers the ribosome binding site of gene *9* preventing its expression.

Analysis of the total nucleic acid content of the kanamycin-resistant synthetic carrier cells by agarose gel electrophoresis revealed a dsRNA migration pattern typical for the phi6 tri-segmented genome confirming the establishment of carrier cells (Fig. S4B). The cell lines were named in reference to their corresponding phi6 genome segment derivatives (Table 1): LM2691(L_kan_, M, S), LM2691(L_kan_, M, S_lys_), LM2691(L, M_kan_, S), LM2691(L, M, S_kan_), LM2691(L_kan_, M, S_TMV_), LM2691(L_kan_, M_TMV_, S), LM2691(L_kan_, M_TMV_, S_TMV_), and LM2691(L_kan_, M, S_Δ(5,9, 12)_). Parallel clones of LM2691(L_kan_, M, S_lys_), LM2691(L, M_kan_, S), and LM2691(L, M, S_kan_) (Fig. S4C) were used in some experiments.

### Quantification of virus load and visualization of phi6 virions and subviral particles within *P. syringae* carrier cells

A recent study of *Escherichia coli* persistently infected by Asterius-like dsDNA phages revealed substantial variation in the phage:host genome ratio between different isolates (41). To assess the quantity of phi6 particles within *P. syringae* carrier cells, we applied quantitative RT-PCR (RT-qPCR) targeting phi6 gene *1*, encoding the major inner capsid protein P1 (42, 43). The P1 copy number in the phi6 polymerase complex is stable (120 copies), and it is produced from the same polycistronic L-segment-specific mRNA as the other components of the polymerase complex (37, 44) (Fig. S1B). Thus, the expression levels of P1 likely correlate well with the particle amounts in the carrier cells.

**Fig 1:**
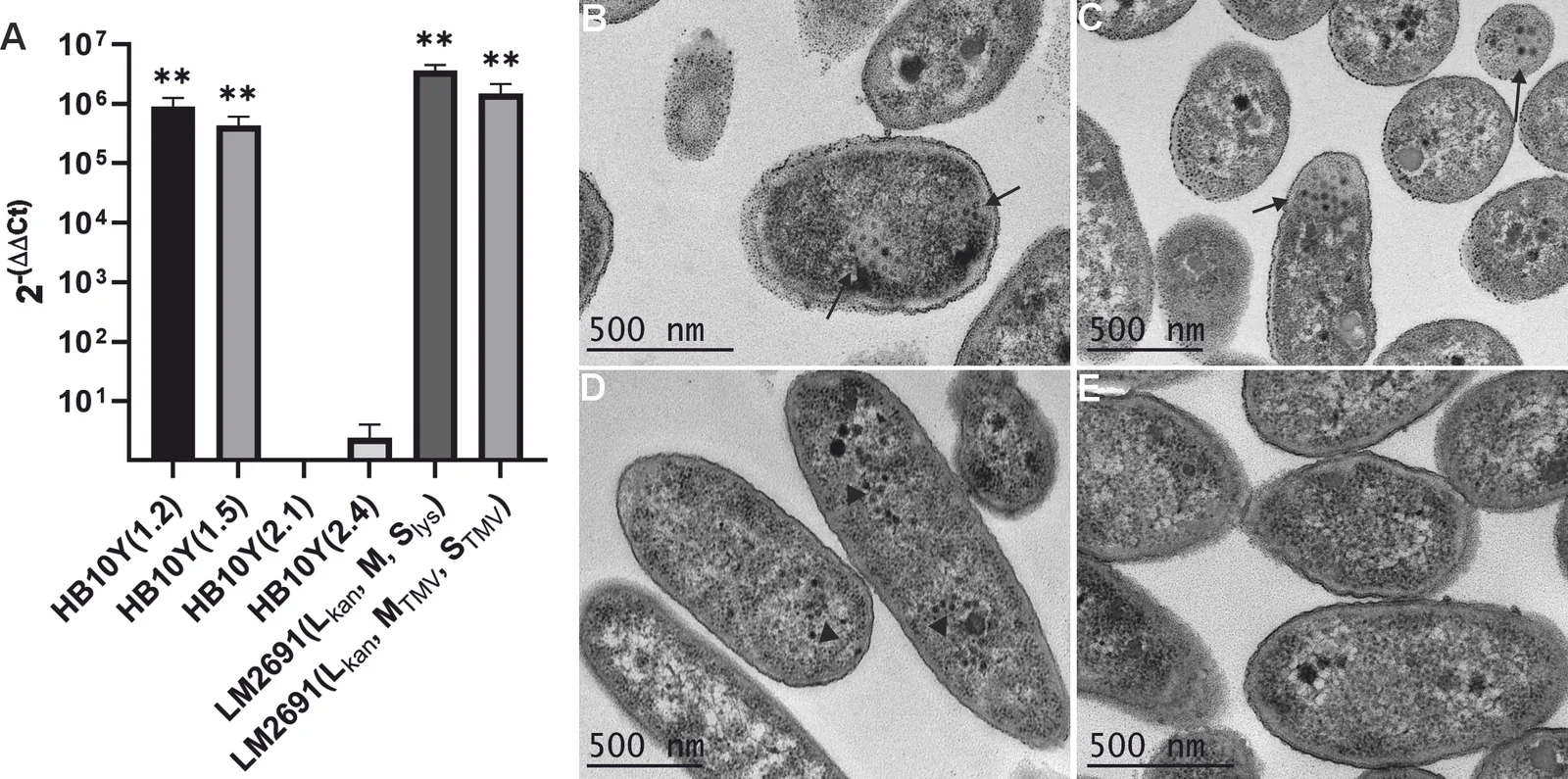
Quantity and organization of phi6 virions and subviral particles within *P. syringae* carrier cells. (**A**) Quantification of phage phi6 gene *1* (encoding the major inner capsid protein P1) expression in *P. syringae* carrier cell lines using RT-qPCR. The mean mRNA expression levels, normalized to the *ftsZ* housekeeping gene of the host, are shown as relative fold change compared to strain HB10Y(2.1) that exhibited the lowest expression level of phi6 gene *1* [highest Ct value; relative quantification (2^−ΔΔCt^) value of 1]. Values represent the mean + standard deviation (SD) of at least two independent biological replicates on a logarithmic scale. **P < 0.01, compared to HB10Y(2.1) (Mann-Whitney test). No amplification of the phi6 gene *1* was detected in the negative controls (see Materials and Methods) included in the RT-qPCR assay or in the samples derived from non-carrier HB10Y verifying the specificity of the used primers. Thin-section transmission electron microscopy of *P. syringae* carrier cell strains HB10Y(1.5) (**B**), LM2691(L_kan_, M, S_lys_) (**C**), LM2691(L_kan_, M_TMV_, S_TMV_) (**D**), and wt HB10Y cells used as a control (**E**). The enveloped, spherical virions are indicated with black arrows and the virion cores with black arrowheads. Cells were fixed in glutaraldehyde and processed for thin section electron microscopy. Scale bars represent 500 nm.

An initial comparison between HB10Y(1.2) and a phi6 lytic infection revealed an almost 30-fold difference in viral gene *1* expression, indicating a substantially higher viral load in cells productively infected by phi6 compared to carrier cell infection (Fig. S5). Further characterization of the different carrier cell isolates revealed considerable differences in gene *1* expression levels between the spontaneous carrier cell lines (Fig. 1A), suggesting major variation in viral loads. HB10Y(2.1) had the lowest and HB10Y(1.2) the highest level of gene *1* expression (over 10^5^-fold difference). Furthermore, gene *1* was highly expressed in the two synthetic carrier cell lines, LM2691(L_kan_, M, S_lys_) and LM2691(L_kan_, M_TMV_, S_TMV_) (Fig. 1A), showing 4.4- and 1.8-fold differences to HB10Y(1.2), respectively. Taking together, gene *1* expression levels were clearly less in all the analyzed carrier cell lines than in the HB10Y host productively infected by phi6 (Fig. 1A; Fig. S5), indicating generally lower virus activity of the carrier cells.

Previous studies have implied that in addition to L-segment, either M- or S-segment is required for the formation of phi6 carrier cells (31). However, the knockout of M- and S-segment coding regions (M_TMV_ and S_TMV_ constructs; Table 1) did not have a major influence on gene *1* expression (Fig. 1A). Thus, while the non-coding regions of M and S involved in the regulation of genome packaging and replication are likely important for the carrier cell formation, none of the structural and nonstructural proteins expressed from M and S are essential.

The genes for all the main phi6 virion proteins are expected to be present in the synthetic carrier cell line LM2691(L_kan_, M, S_lys_) and in the spontaneous carrier cells. Accordingly, enveloped, spherical phi6 virions were identified in thin section transmission electron microscope (TEM) images of these cells (Fig. 1B and C). Knockout of the M- and S-segment genes prevents the production of outer capsid and envelope, and therefore phi6 virion cores (i.e., polymerase complexes enclosing the viral dsRNA) were observed in TEM images of LM2691(L_kan_, M_TMV_, S_TMV_) (Fig. 1D). Viral particles were not observed in HB10Y cells (Fig. 1E).

### Persistent phi6 infection has no major effect on host growth at optimal temperature of 28°C

**Fig 2:**
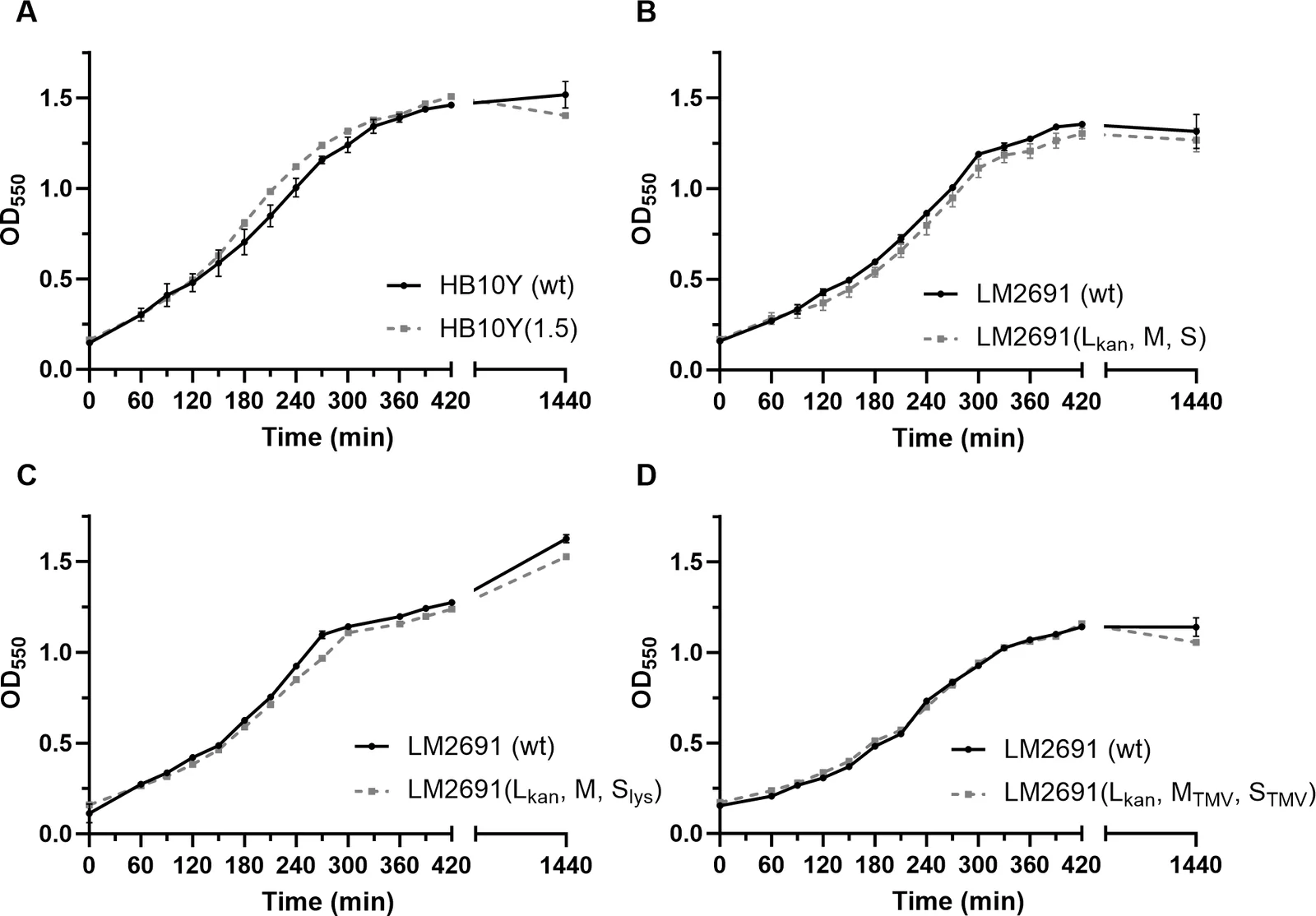
Impact of persistent phi6 infection on *Pseudomonas syringae* growth in LB medium at 28°C. Growth rate of spontaneous carrier cell line HB10Y(1.5) compared to wild-type (wt) HB10Y (**A**); growth rate of synthetic carrier cell lines LM2691(L_kan_, M, S) (**B**), LM2691(L_kan_, M, S_lys_) (**C**), and LM2691(L_kan_, M_TMV_, S_TMV_) (**D**) compared to wt LM2691. Bacteria were cultivated in LB medium at 28°C, and the optical density was measured at specific time points over a 24-h period. Mean optical density at a wavelength of 550 nm (OD_550_) ± standard error of the mean (SEM) of at least three replicates is presented.

To analyze the possible burden the persistent phi6 infection may inflict upon the host, the growth patterns of four spontaneous (Fig. 2A; Fig. S6) and three synthetic carrier cell lines (Fig. 2B through D) were determined and compared to the corresponding wt strains (HB10Y and LM2691, respectively). Based on the growth curve analysis, persistent phi6 infection does not notably affect the growth rate of the *P. syringae* when cultivated in Luria-Bertani (LB) medium at 28°C (optimal temperature for *P. syringae* [45]).

### Persistent phi6 infection can have different phenotypic effects on the host depending on the temperature

**Fig 3:**
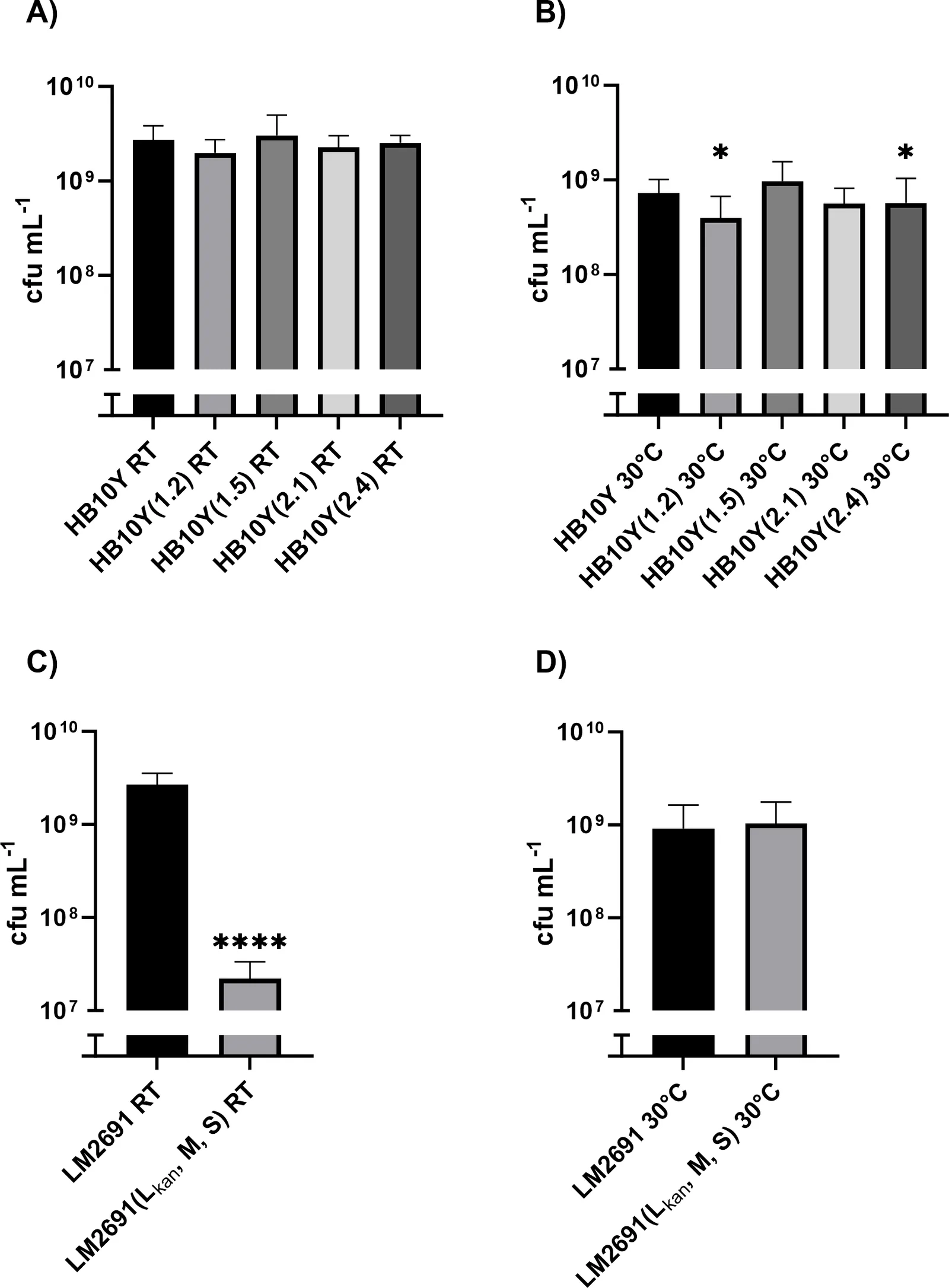
Impact of persistent phi6 infection on *Pseudomonas syringae* host cell density. Cell density (CFU mL^−1^) was determined after 24-h cultivation in LB at RT (**A and C**) or 30°C (**B and D**) and plotted on a logarithmic scale. (**A and B**) Cell densities of spontaneous carrier cell lines HB10Y(1.2), HB10Y(1.5), HB10Y(2.1), and HB10Y(2.4) are compared to wt HB10Y; (**C and D**) cell density of synthetic carrier cell line LM2691(L_kan_, M, S) is compared to wt LM2691. Values represent the mean + SD of at least three independent biological replicates. ****P < 0.0001; *P < 0.05, compared to corresponding wt strain (HB10Y or LM2691) (Mann-Whitney test).

The potential effects of global warming on the spread of viruses and diseases have been recognized. However, climate change may also modify the ways viruses interact with their hosts. Therefore, the possible effect of temperature on the phi6 carrier cells was analyzed after cultivation at room temperature (RT; 22°C) or 30°C. Instead of measuring optical density (turbidity), which does not distinguish between dead and living cells, we used serial dilution plating to more accurately measure the concentration of viable cells. RT is the optimal temperature for phage phi6 propagation, whereas previous literature suggests that *P. syringae* growth is largely unaffected at a temperature range of 25°C to 33°C (45). We observed some decrease in *P. syringae* cell densities at 30°C compared to RT (Fig. 3A and B), regardless of the carrier cell status. Nevertheless, in line with the optical density data (Fig. 2A; Fig. S6), the cell densities of the spontaneous carrier cell cultures at RT did not differ significantly from the corresponding control cultures (Fig. 3A). However, at 30°C, two out of four spontaneous carrier cell lines showed minor (21%–45% reduction) but nevertheless significant drop in cell density compared to wt HB10Y (Fig. 3B). Thus, in addition to the variation in virus load (Fig. 1A), the responses of spontaneous carrier cell lines to cultivation temperature can vary. Notably, the synthetic carrier cell line LM2691(L_kan_, M, S) had a considerably lower cell density compared to LM2691 when cultivated at RT (Fig. 3C) (two orders of magnitude reduction), whereas no difference was observed when the strains were grown at 30°C (Fig. 3D).

### Stability of the phi6 carrier cell interaction varies at different temperatures

**Fig 4:**
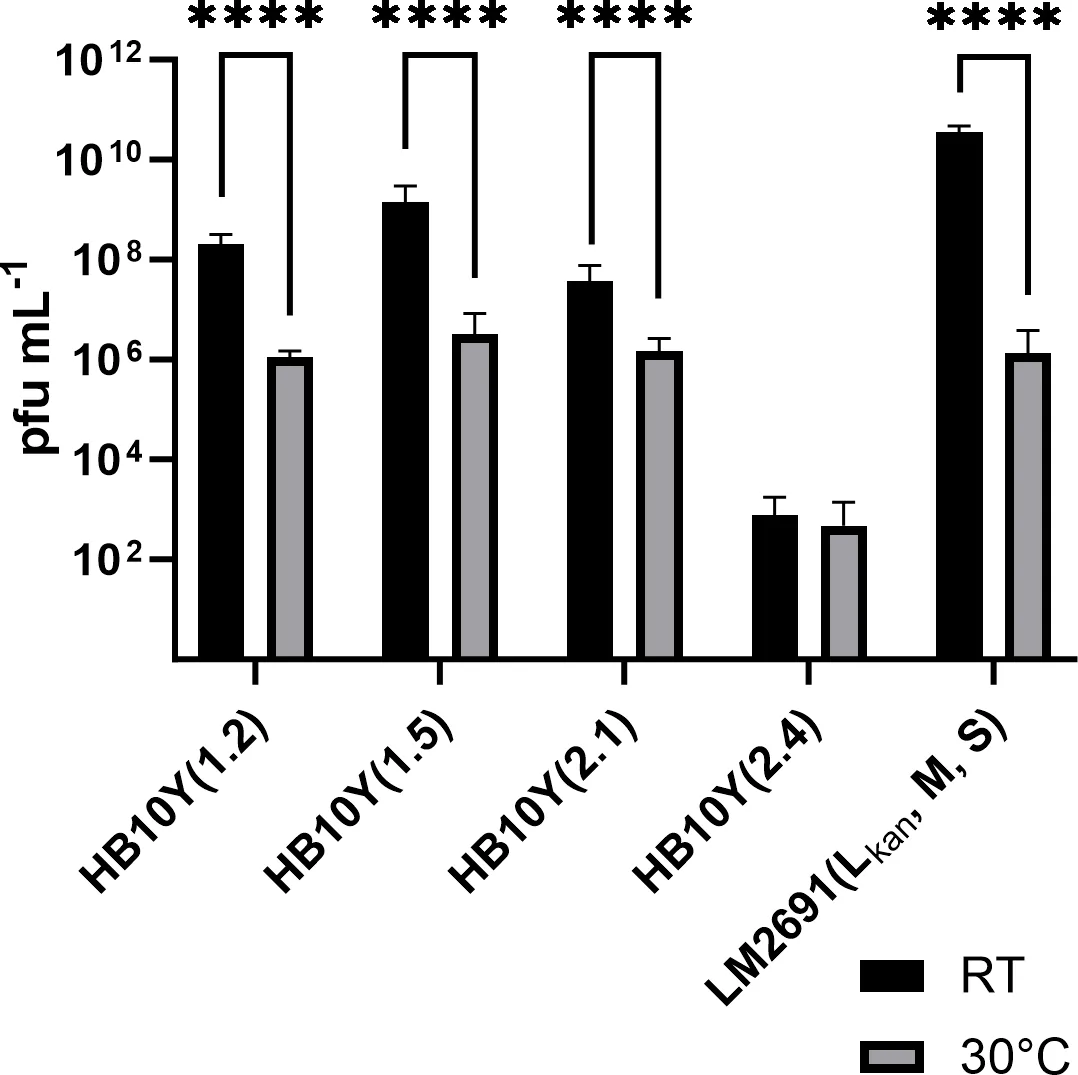
Effect of temperature on the stability of phi6 carrier cell interaction with its *Pseudomonas* host. Number of phi6 phages (pfu mL^−1^) liberated by phi6 carrier cell strains during 24-h cultivation in LB broth at RT (black bars) and 30°C (gray bars). Values represent the mean + SD of at least three independent biological replicates, plotted on a logarithmic scale. ****P < 0.0001 (Mann-Whitney test).

To understand the underlying reason for the significantly reduced growth of the synthetic carrier cell line LM2691(L_kan_, M, S) at RT (Fig. 3C) but not at 30°C (Fig. 3D), we assayed the stability of the carrier state interaction at the different temperatures by measuring the number of phages liberated in the medium. At 30°C, the phage titers in the LM2691(L_kan_, M, S) culture supernatants were significantly lower than at RT (over four orders of magnitude reduction; Fig. 4). To better understand the underlying reason for this observation, we excluded the possibility that the overall phi6 infectivity is reduced due to exposure to 30°C (Table S2). Nevertheless, we observed a sixfold difference in the average burst size of phi6 between the two temperatures (183 ± 37 at RT and 29 ± 5 at 30°C; Fig. S7). However, this difference in the burst size can only explain a small fraction of the observed, over four orders of magnitude, difference in the number of infectious phi6 phages in the LM2691(L_kan_, M, S) culture supernatant at RT and 30°C indicating that additional factors contribute to this phenomenon. Together, the observed significant reduction in cell density (Fig. 3C) and increase in infectious phi6 phages in the culture supernatant at RT (Fig. 4) both suggest more frequent activation of the lytic, productive infection when LM2691(L_kan_, M, S) is cultivated at RT than at 30°C. In other words, entry of phi6 carrier cells to lytic infection is less frequent at 30°C, i.e., the carrier cell interaction is stabilized at elevated temperature.

A similar phenomenon was observed when the culture supernatants of the spontaneous carrier cell lines grown at RT or 30°C were analyzed (one to two orders of magnitude titer difference between the temperatures; Fig. 4). The only exception was HB10Y(2.4), in which case the measured phage titers were generally low and the titer difference between RT and 30°C cultures was not statistically significant (Fig. 4). Furthermore, similar results for all the tested carrier cell strains were obtained when cells were grown in M9 minimal medium (instead of LB) at RT or 30°C (Fig. S8).

As the effect of temperature was more dramatic on the tested synthetic carrier cell line LM2691(L_kan_, M, S) than on the spontaneous carrier cell strains (Fig. 4), we further extended the temperature analyses assays to two additional synthetically established phi6 carrier cell types: LM2691(L, M_kan,_ S) and LM2691(L, M, S_kan_) (Fig. S9) which have the kanamycin resistance gene in M- or S-segment, respectively, instead of L-segment, and to an additional clone of LM2691(L_kan_, M, S). The tested two clones of LM2691(L, M_kan,_ S) and the clone 2 of LM2691(L_kan_, M, S) all showed one to two orders of magnitude reduction in phage titer at 30°C (Fig. S9), resembling the phenotype of the spontaneous carrier cell strains (Fig. 4). Interestingly, the opposite was observed for the two clones of LM2691(L, M, S_kan_) cultures: the phage titer was one order of magnitude higher at 30°C than at RT (Fig. S9). These results demonstrate that the impact of temperature on phi6 infection strategy (lytic vs carrier cell) is not uniform but can vary between strains. Nevertheless, the majority of the tested carrier cell lines reacted to the temperature in varying degrees.

Previous studies indicate that phi6 lytic enzyme P5 loses activity at temperatures above 20°C (46). Thus, the stabilization of the carrier cell interaction at elevated temperatures could result from temperature inactivation of the viral lytic enzyme. To test this hypothesis, chloroform-treated HB10Y cells were incubated with phi6 P5 at RT or 30°C (Fig. S10). The turbidity of the cell suspension reduced in the presence of P5, but we were unable to detect any major difference in the lytic activity of P5 between the two temperatures. It is therefore likely that there are other mechanisms that contribute to the observed temperature-induced effects of the phi6 carrier cells.

### Phi6 carrier cells show resistance to secondary phi6 infection

**Fig 5:**
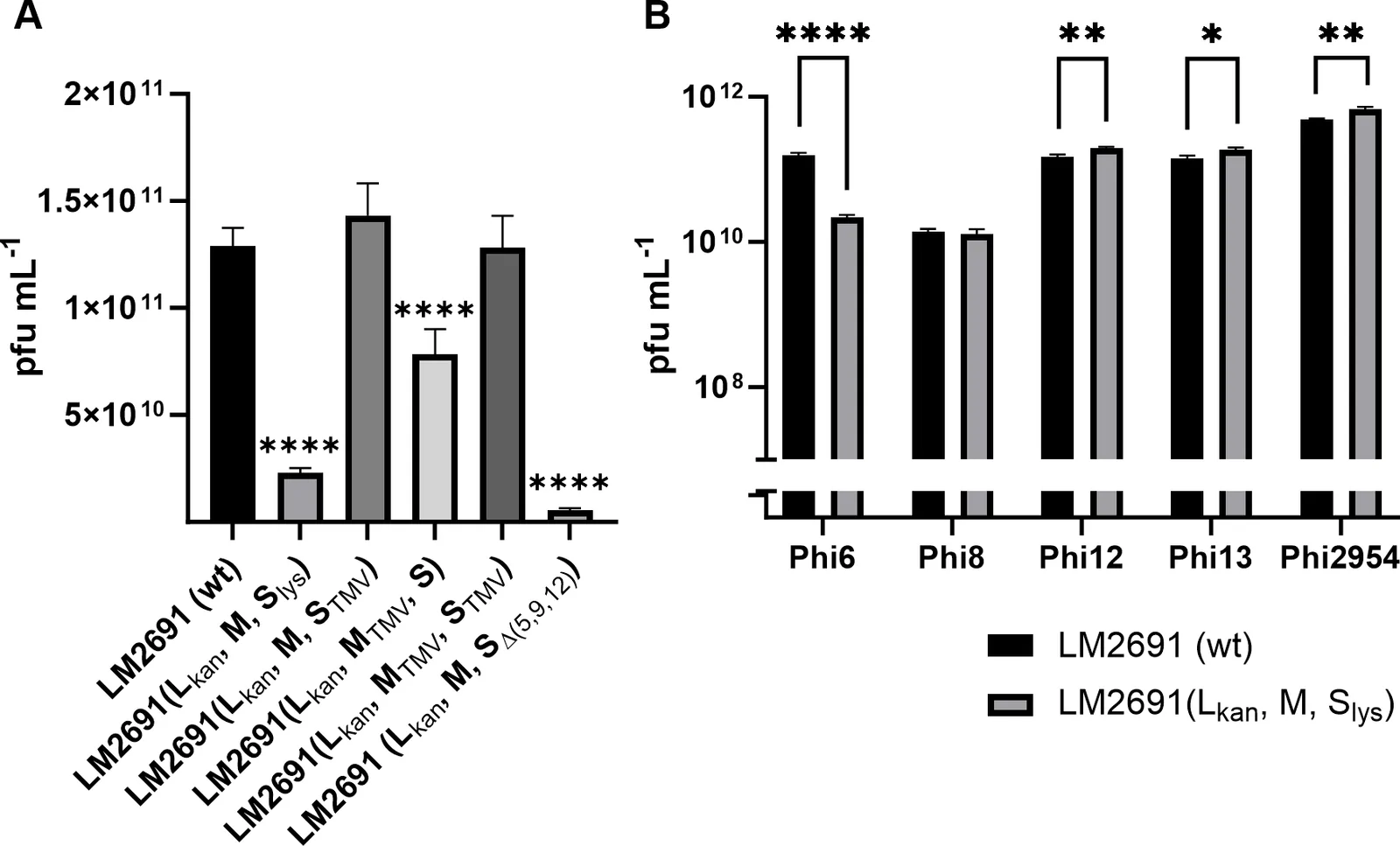
Sensitivity of phi6 carrier cells to secondary cystovirus infections. (**A**) Sensitivity of knockout carrier cell lines to secondary phi6 infection. Phi6 infectivity (titer; y-axis in a linear scale) against the wt LM2691 and the phi6 knockout carrier cell lines LM2691(L_kan_, M, S_lys_), LM2691(L_kan_, M, S_TMV_), LM2691(L_kan_, M_TMV_, S), LM2691(L_kan_, M_TMV_, S_TMV_), and LM2691 (L_kan_, M, S_∆(5,9,12)_). (**B**) Cystovirus infectivity (titer; y-axis in logarithmic scale) against the wt LM2691 (black bars) and the carrier cell line LM2691(L_kan_, M, S_lys_) (gray bars). The mean + SEM of at least three biological replicates is presented. ****P < 0.0001; **P < 0.01; *P < 0.05 compared to wt LM2691 (Mann-Whitney test).

Spontaneous phi6 carrier state cultures were initially identified when properties of phi6 resistant bacteria, obtained after exposure of the host to phi6 infection, were analyzed (13, 15). This strategy was also used in this study. To obtain more direct evidence that these two phenomena, the carrier cell formation and the phage resistance, are linked, we took advantage of the synthetic carrier cell line *P. syringae* LM2691(L_kan_, M, S_lys_). Due to the defective viral lytic enzyme, LM2691(L_kan_, M, S_lys_) does not release phage particles, facilitating the interpretation of the results. In line with the earlier observations on spontaneous carrier state cultures (15, 30), LM2691(L_kan_, M, S_lys_) showed significantly reduced susceptibility to secondary phi6 infection compared to the non-carrier host strain (Fig. 5A). However, a low number of infectious viral particles were detected in the LM2691(L_kan_, M, S_lys_) culture supernatant after secondary phi6 infection. These viruses likely represent progenies of the wt phi6 which have been able to productively, albeit less efficiently, infect the carrier cells. Alternatively, the wt lytic enzyme of the entering virus has complemented the defective lysis gene of the carrier cell virus enabling production and release of infectious progenies.

### Gene *8* of phi6 S-segment is required for carrier cell-induced superinfection exclusion

To pinpoint the genetic basis of the carrier cell-induced superinfection exclusion, we produced synthetic carrier cells using S- and M-segment knockout constructs (S_TMV_ and M_TMV_, respectively; Table 1), and the resulting three strains were subjected to secondary phi6 infection (Fig. 5A). A significant reduction in phi6 titer (approximately 39%) was observed on strain LM2691(L_kan_, M_TMV_, S) compared to LM2691, whereas strains LM2691(L_kan_, M, S_TMV_), LM2691(L_kan_, M_TMV_, S_TMV_), and LM2691 equally supported phi6 infection. Thus, the strains harboring the S-segment knockout construct (S_TMV_) supported phi6 infection better than the strains expressing wt S-segment. Both constructs contain the S-segment-specific 5′ packaging and 3′ replication signals. All together, these results indicate that the protein coding region of phi6 S-segment contributes to the immunity against secondary infections and provide additional evidence that the superinfection exclusion and carrier cell phenomena are interconnected.

To identify more precisely the gene(s) responsible for superinfection exclusion, phi6 was titrated against synthetic carrier cell line LM2691 (L_kan_, M, S_∆(5,9,12)_) harboring an S-segment deletion construct (S_∆(5,9,12)_; Table 1). This construct encodes phi6 major outer capsid protein P8 (gene *8*), whereas all other genes of the segment (Table 1) have been knocked out. The phi6 titer was 96% lower on LM2691 (L_kan_, M, S_∆(5,9,12)_) than on LM2691 (Fig. 5A), showing that the phi6 gene *8* contributes to the superinfection exclusion.

### Phi6 carrier cell infection does not confer resistance against secondary infections of related phages

Carrier state interaction protected the host from superinfection by the same virus (Fig. 5A), but could the pre-existing virus prevent secondary infections by related viruses? To analyze this, we tested the susceptibility of the synthetic phi6 carrier cell line *P. syringae* LM2691(L_kan_, M, S_lys_) to cystoviruses phi8, phi12, phi13, and phi2954. No significant difference was detected in the susceptibility of the carrier cell and wt strains to phi8, which is a distant relative of phi6 (47). Interestingly, infection of the phi6 carrier cells by the other cystoviruses resulted in a small (32%–39%), but nevertheless significant increase in the phage titers when compared to infection of the non-carrier strain (Fig. 5B). These data suggest that carrier cell infection confers resistance (i.e., superinfection exclusion) against secondary infections of the same phage (phi6), but the protective effect does not extend to related viruses (Fig. 5).

### P8 of phi6 suppresses phi6 but not phi13 transcription

**Fig 6:**
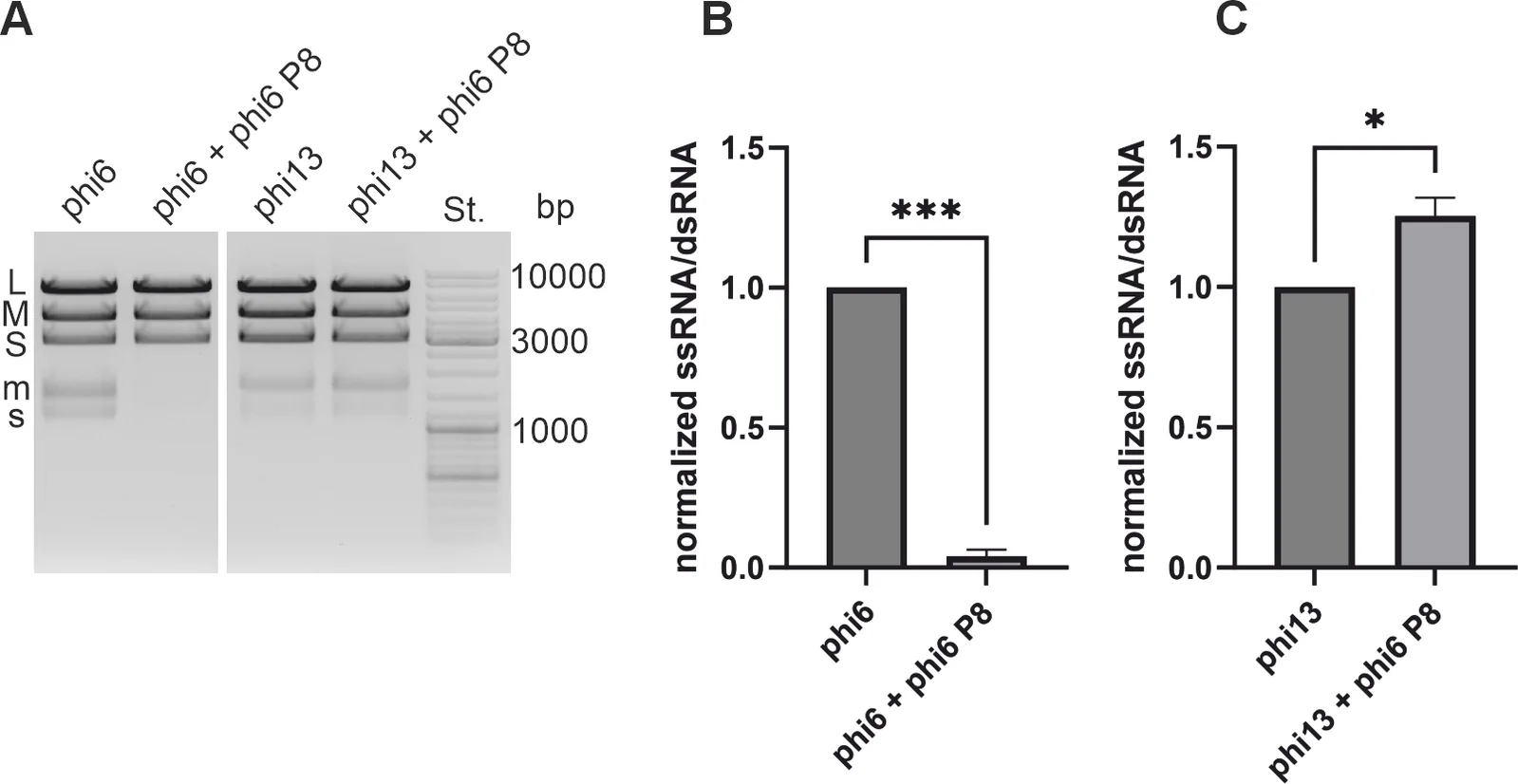
The effect of phi6 P8 on the transcription activity of phi6 and phi13 NCs. (**A**) Agarose gel electrophoresis analysis of reaction products from *in vitro* transcription assays of phi6 and phi13 NCs, with or without phi6 protein P8. The mobility of the double-stranded (uppercase letters) and single-stranded (lowercase letters) RNAs is indicated. The sizes of selected bands of the GeneRuler DNA Ladder Mix (Thermo Scientific) molecular size standard (St.) are indicated in base pairs (bp). The relative transcription activity of phi6 (**B**) and phi13 (**C**) NCs was calculated from the quantitated intensities of the ssRNA and dsRNA bands and plotted as the ssRNA/dsRNA ratio. The ssRNA/dsRNA ratio values of the control reaction without P8 were used for normalization. Error bars represent standard deviations of the means for three replicates. Statistically significant differences between the control reactions and reactions with P8 are indicated (***) *P* < 0.001, (*) *P* < 0.05 (unpaired *t*-test with Welch’s correction).

The outer layer of the phi6 NC, made of protein P8, must be disassembled to enable the expansion of the inner capsid, which is necessary for transcriptional activation (48). We hypothesize that the presence of the outer capsid protein P8 in phi6 carrier cells could disturb this essential disassembly process by shifting the balance from P8 shell disassembly toward assembly, resulting in the stabilization of the double-layered NC of the superinfecting phage. This would thereby prevent transcriptional activation of the entering virus and result in superinfection exclusion. To test this hypothesis, we used an *in vitro* transcription assay based on purified phi6 NCs. Two parallel reactions were established, with and without purified P8 of phi6 (Fig. 6A). The reaction products were analyzed by electrophoresis in native agarose gel, which allows separation of the transcription products (ssRNAs) from the genomic dsRNAs (Fig. 6A). In the conditions used, the phi6 NC transcribes the M- and S-segments, and the corresponding ssRNAs were detected in the EtBr-stained gels. However, the transcription activity was significantly reduced (96% reduction, *P* < 0.001; Fig. 6B) if the reactions were carried out in the presence of phi6 P8, showing that excess of soluble outer capsid protein can efficiently prevent the intra-capsid transcription process of phi6.

The superinfection exclusion experiments (Fig. 5) suggested that P8-mediated infection inhibition is species-specific. Therefore, *in vitro* transcription experiments were conducted also with phi13 NCs. *In vitro* transcription by phi13 NCs was confirmed both in the absence and presence of phi6 P8 (Fig. 6A), showing that P8 from a heterologous origin does not suppress phi13 transcription. Unexpectedly, the transcription activity of phi13 NCs was slightly increased in the presence of phi6 P8 (25%, *P* < 0.05; Fig. 6C) compared to the control with no P8. This increase is likely due to EGTA from the P8 storage buffer (approximately 0.05 mM in the reaction) rather than P8 itself, as EGTA can chelate Ca²^+^ and promote P8 shell disassembly, thereby activating transcription (37). Taken together, excess of phi6 P8 strongly and specifically inhibits phi6 NC transcription, consistent with a model in which P8 interferes with NC uncoating that is a prerequisite for transcriptional activation of the entering phage during superinfection.

### Carrier cells are potential incubators for new viral variants

The pre-established phi6 infection appeared to enhance phage production following a secondary infection with cystoviruses phi12, phi13, or phi2954 (Fig. 5B). To better understand this phenomenon, we characterized the progeny phages produced by the phi6 carrier cells infected by phi13. The resulting lysates were plated on *P. syringae* LM2489, which can be infected by both phi6 and phi13, and on HB10Y, the natural host of phi6 that is resistant to phi13. Plaques were obtained on LM2489 but also in lower frequency on HB10Y. Formation of plaques on HB10Y suggests that (i) the secondary infection of phi6 carrier cells by phi13 results in reactivation of phi6 infection by providing a functional lysis gene *5* from phi13 (through S-segment recombination or reassortment) or that (ii) genes encoding the host recognition proteins in phi13 M-segment have been replaced by phi6 host recognition gene *3,* providing the progenies phi6 host specificity. To test these hypotheses, we analyzed the genotypes of four progenies obtained on HB10Y by RT-PCR using primers specific for the phi6 M-segment (host recognition gene *3*), phi13 S-segment (lysis gene *5*), and phi13 L-segment (inner capsid protein gene *1*). All four isolates appeared to be hybrids containing phi13 S-, phi6 M-, and phi13 L-segments-specific sequences (Fig. S11A, B, and E), supporting the latter hypothesis. To distinguish between the possibility of phi13 acquiring phi6 gene *3* via recombination or replacement of the entire M, we analyzed the hybrids by using additional phi6 M-segment-specific primers. In addition to gene *3*, all hybrids were positive for phi6 genes *6* and *10* (encoding envelope-associated proteins; Fig. S11C and D). This observation highlights the role of carrier cells as a potential source of new virus variants via reassortment of genome segments.

## DISCUSSION

Persistent viral infections appear to be more abundant and ubiquitous in nature than previously recognized. However, understanding of the mechanistic basis and ecological implications of such long-term virus-host interactions has remained limited largely due to the lack of stable carrier cell model systems. Here, we utilized dsRNA bacteriophage phi6 to elucidate the alternative persistent infection strategy of dsRNA viruses, especially those infecting bacteria. By using reverse genetics to establish stable lysis-defective carrier cells and viral gene knockouts, we analyzed the effects of carrier cell interaction to the host and to superinfections and identified environmental factors favoring viral persistence.

Previous studies found no significant growth rate difference between (spontaneous) phi6 carrier cell and wt non-carrier cultures or identified a negative correlation between phi6 carrier cell status and host cell growth (13, 15). Our results show that persistent phi6 infection does not influence host growth under conditions in which the carrier cell interaction is stable (Fig. 2 and 3). Interestingly, Cai et al. (12) reported a positive effect of persistent dsRNA phage phiNY infection on *Microvirgula aerodenitrificans* growth, implying a mutualistic relationship. These observations suggest that persistent dsRNA phage infections can elicit different phenotypic changes in the host resembling the cryptic and beneficial effects described for persistent fungal dsRNA virus infections (49).

We showed that phi6 carrier cell lines reacted to the growth temperature in varying degrees. Results on several different strains indicated stabilization of the carrier cell interaction at elevated temperature, i.e., reduction of the entry of phi6 into the lytic productive cycle (Fig. 4). Nevertheless, some phi6 carrier cell lines also responded to the temperature increase in the opposite way; temperature increase apparently promoted the entry to the lytic cycle (Fig. S9). The latter phenotype resembles that described for some DNA phages that are lytic in warm conditions but establish either lysogeny (50, 51), pseudolysogeny (52), or carrier state (53) at lower temperatures. The observed phenotypic diversity among phi6 carrier cells likely promotes adaptation of cystovirus populations to changing environments by enabling maintenance of both intra- and extracellular reservoirs of phages under different environmental conditions. The ability of a phage to change its infection strategy as a result of a relatively modest temperature change is interesting in the context of global warming. The temperature dependence of the phage-host interaction dynamics may have profound implications in the ecology of microbial communities and consequently ecosystem functioning. Furthermore, changes in the infection strategy of phages of plant pathogenic bacteria, such as phi6, at elevated temperatures are also of great agricultural relevance.

The spontaneous carrier cell isolates varied considerably in terms of viral load (Fig. 1A). This could reflect pre-existing genetic diversity in the host or the phage, or different evolutionary trajectories of the carrier cells. Identifying the genetic basis underlying these phenotypic differences would be interesting. However, because cystoviruses are RNA viruses and therefore accumulate mutations readily (roughly 10⁻³ to 10⁻^6^ errors per nucleotide per replication) (54), there could be tens or hundreds of distinct mutation sites among phages from different carrier cell strains. Determining the phenotypic effects of each mutation would require introduction of individual mutations into the viral genome using reverse genetics, followed by verification of the resulting viral sequences, and subsequent phenotypic analyses. Consequently, we consider a detailed genetic comparison of phages from different carrier cell lines to be beyond the scope of this study, but it represents an intriguing direction for future research.

Autoinhibitory effect of phi6 carrier cells has been previously reported (13, 15, 30), and persistent infections of animal and plant dsRNA viruses of the order *Reovirales* also show resistance to secondary infections (6, 55). To further explore the superinfection exclusion of persistent phi6 infection, we utilized synthetic lysis-defective carrier cell line LM2691(L_kan_, M, S_lys_) that does not release phage, allowing for more controlled analysis of the phenomenon. Our results show that the intracellular phage provides a protective benefit for the host cell against the same phage (Fig. 5A) but does not confer resistance against related cystoviruses (Fig. 5B), demonstrating that the superinfection exclusion is selective. In fact, the phi6 carrier cells were somewhat more susceptible to infections of cystoviruses phi12, phi13, and phi2954 than wt non-carrier strain, suggesting a synergistic interaction. However, the same phenomenon was not observed with cystovirus phi8, as the phi6 carrier and wt cells were equally susceptible to phi8 infections, possibly reflecting the distant relatedness of these cystovirus species (47). These observations are relevant for the development of cystovirus-based biocontrol of *P. syringae* infections in crops and suggest that cocktails of distantly related cystoviruses should be utilized to maximize the agricultural benefit.

Superinfection exclusion has been reported for various viruses, including important pathogens of humans, animals, and plants (56), but the underlying mechanisms, such as viral determinants, remain poorly understood. Results from the gene knockout assays showed that gene *8,* encoding the major outer capsid protein, is the major genetic contributor of the autoinhibitory effect (Fig. 5A). Interestingly, a similar phenomenon was recently described for rice black-streaked dwarf virus (RBSDV), a member of the dsRNA virus family *Spinareoviridae*, order *Reovirales* (55). The outer capsid protein of RBSDV was shown to make the plant host less susceptible to infections of RBSDV and a related virus (southern black-streaked dwarf virus) but more susceptible to an unrelated virus (rice stripe virus). However, the underlying mechanism of the phenomenon is not understood. Notably, the proteins contributing to superinfection exclusion in spinareovirus and cystovirus both form an icosahedral T = 13 lattice (57–59) and may thus share a common evolutionary origin. In phi6, the outer capsid of the entering virus needs to be disassembled to allow transcriptional activation of the inner capsid (48), essential for the establishment of a productive infection cycle. By using controlled *in vitro* experiments, we could demonstrate that P8 prevents transcription by phi6 NC (Fig. 6). The excess of soluble P8 likely disrupts P8 shell disassembly and transcriptional activation of the entering virus by shifting the balance from disassembly toward P8 shell assembly, which results in superinfection exclusion. This would be analogous to what has been described for tobacco mosaic virus (TMV), a filamentous ssRNA virus, where excessive production of the viral coat protein protects the host cell from secondary TMV infections, presumably by inhibiting the disassembly of the challenge virus (60).

To elucidate the evolutionary potential of persistent dsRNA virus infections, we characterized progeny phages produced by lysis-defective phi6 carrier cells following secondary phi13 infection (Fig. S11). The results suggest infrequent reassortment events in which the M-segment of phi13 is replaced by the M-segment of the persistent phi6 phage, providing the progeny phages the phi6 host specificity. This observation demonstrated the potential of carrier cells to generate new virus variants via reassortment. Previous studies on natural cystovirus isolates have indicated reassortment events (61), and co-infection of cells with phi6 mutants or *lacZ-*tagged phi6 variants has also been shown to result in reassortment (62, 63). Here, we demonstrate that in addition to co-infection (62, 63), superinfection of persistently infected hosts can promote the formation of new viral variants representing crosses between two viral species from two different virus genera (*Orthocystovirus* and *Gammacystovirus*). This is potentially an important mechanism by which dsRNA viruses with segmented genomes, such as rotaviruses, reoviruses, orbiviruses, and phytoreoviruses, evolve and by which defective viruses persistently infecting their host may be recovered.

This study provides insights into the mechanistic basis and ecological implications of persistent dsRNA virus infections. We demonstrated that dsRNA phage phi6 switches infection strategy between lytic and carrier cell infection depending on temperature. We also identified a viral determinant involved in the autoinhibitory effect of the phi6 carrier cells, thus contributing to the knowledge of superinfection exclusion. The reverse genetic systems developed for other dsRNA viruses (64–69) could enable elucidation of the carrier cell phenomenon in eukaryotic hosts and provide further insights into the persistent infection and its evolution among dsRNA viruses. Furthermore, the ability of the carrier cells to function as incubators of new viral variants warrants further studies. Our results contribute to the understanding of dsRNA phage infection dynamics, which will support the design of cystovirus-based disease control strategies in agriculture.

## MATERIALS AND METHODS

### Bacterial strains and phages

*P. syringae* pv. *phaseolicola* strain HB10Y is the host for cystovirus phi6 (species *Orthocystovirus phi6*) (70). HB10Y is sensitive to cystovirus phi2954 (species *Deltacystovirus phi2954*) (71) but resistant to cystoviruses phi8, phi12, and phi13 (species *Alphacystovirus phi8*, *Betacystovirus phi12,* and *Gammacystovirus phi13*, respectively) (14). *P. syringae* LM2489, a rough derivative of HB10Y, was used as the host for phi8, phi12, and phi13 (14). *P. syringae* LM2691 (31) carries plasmid pLM1086, which constitutively expresses T7 RNA polymerase, and was used to establish synthetic phi6 carrier cells. LM2691 is sensitive to all phages used in this study. *Escherichia coli* DH5α was used for plasmid propagation (see Table 1 for the plasmids). Bacteria were grown in LB broth or minimal medium, supplemented with kanamycin (25 µg mL^−1^) when necessary. The optical density of liquid cultures was measured using Clormic P Selecta spectrophotometer.

Bacteriophages were propagated on LB agar plates (1% [wt/vol]) using the standard double agar layer technique (72), LB soft agar (0.7% [wt/vol]) for plating, and the plates were incubated overnight at RT. High-titer virus stocks were prepared by collecting top agar from plates showing semi-confluent lysis of the bacterial lawn by the phage (73). Liquid cultures in LB medium were used to produce phi6 and phi13 phages for virion purification (73, 74).

### Isolation of synthetic and spontaneous phi6 carrier cell lines

For the generation of synthetic phi6 carrier cell lines, electroporation-competent *P. syringae* LM2691 cells were prepared (31), mixed with phi6 segment-specific plasmid constructs (Table 1; 200 ng of each plasmid/100 µL of cells), and incubated on ice for 10 min before electroporation (25 μF, 2.5 kV, 200 Ω; Bio-Rad MicroPulser). Electroporated cells were recovered by incubation in 1 mL of SOC solution (32) at RT with agitation for 2 h and plated onto kanamycin-containing plates. After incubation at RT for 2 days, individual kanamycin-resistant colonies were picked and purified by two successive colony isolations.

Spontaneous phi6 carrier cells were isolated as previously described (15). Approximately 1 × 10^9^ pfu of phi6 phage was mixed with 1 × 10^8^ HB10Y cells, plated, and incubated at 26°C for 2 days. Individual phi6-resistant colonies were picked and purified twice by single colony isolation.

For the initial screening, phi6 carrier cells grown in liquid cultures were collected by centrifugation, disrupted by repeated freeze (−20°C)-thaw cycles, and the released total nucleic acid content of the cells was analyzed by electrophoresis in 1% (wt/vol) agarose gel in Tris-acetate-EDTA (TAE) buffer containing 0.5 µg mL^−1^ ethidium bromide and imaged (ChemiDoc Touch Imaging System, Bio-Rad). Intracellular phi6 in the spontaneous carrier cells was confirmed by RT-PCR amplification of viral sequences (see below). For this, total cellular RNA was extracted using phenol-chloroform extraction (TRIsure, Meridian Bioscience), followed by sodium acetate precipitation.

### cDNA synthesis and RT-PCR

Total cellular RNA was extracted from concentrated suspensions of overnight-grown liquid bacterial cultures (non-infected or infected by lytic phi6) by phenol-chloroform extraction (32) or commercial kits. For RT-qPCR, extracted RNA (isolated with GeneJet RNA purification kit, Thermo Scientific) was treated with DNase RQ1 (Promega) to remove genomic DNA. RNA concentration was measured using a NanoDrop 2000 UV-Vis spectrophotometer. Extracted RNA (~0.5 µg) was reverse transcribed using random hexamer primers and SuperScript IV Reverse Transcriptase (Invitrogen Life Technologies). The resulting first-strand cDNA was PCR amplified with Phusion High-Fidelity DNA Polymerase (Thermo Scientific), and the products analyzed by agarose gel electrophoresis. Alternatively, SYBR green real-time qPCR was performed on the QuantStudio Real-Time PCR system (Applied Biosystems; primers in Table S1). The expression data were normalized using the *ftsZ* gene of the host as the reference gene. Relative gene expression was analyzed using the 2^−ΔΔCt^ method (75). Negative controls without the cDNA template or reverse transcriptase were included in each RT-qPCR assay. In addition, RNA extracted from non-carrier wt HB10Y cells was subjected to the RT-qPCR experiment.

### Electron microscopy

Overnight-grown bacterial cells were collected, fixed with 2.5% (vol/vol) glutaraldehyde (in 20 mM KPO_4_, 1× LB broth) at RT for 1 h, and washed three times with 20 mM KPO_4_. The glutaraldehyde-fixed cell pellet was treated with osmium tetroxide, dehydrated, embedded in Epon resin, thin-sectioned, and post-stained with uranyl acetate by the Electron Microscopy Unit of the University of Helsinki. The thin layers were examined using transmission electron microscopy (TEM; Jeol JEM-1400, 80,000 V).

### Secondary infection of phi6 carrier cells by phi13

LM2691(L_kan_, M, S_lys_) cells grown in liquid culture to exponential growth phase were infected by phi13 at a multiplicity of infection of 5. Forty minutes post-infection, unbound phages were removed by centrifugation (4,600 × *g*, 1 min; Thermo Scientific Sorvall Centrifuge, F20 rotor) and the cell pellet resuspended in fresh LB medium. This procedure was repeated three times and culturing then continued overnight. Resulting lysates were plated on LM2489 and HB10Y. Individual plaques were isolated from HB10Y lawn and used to produce high-titer lysates. RNA was extracted from the lysates using PureLink Viral RNA/DNA MiniKit (Invitrogen) for RT-PCR.

### *In vitro* transcription assays

Phi6 and phi13 NCs were obtained from the purified phi6 and phi13 virions, respectively, via removal of the envelope using detergent treatment (59, 73, 74). Uncoating of phi6 NCs was triggered using EDTA and the released P8 purified as previously described (73). *In vitro* NC transcription assays were performed in 50 mM Tris-HCl (pH 8.0), 50 mM NH₄Ac, 150 mM KCl, 5 mM DTT, 3 mM MnCl₂, 1 mM MgCl₂, 0.01 mM CaCl₂, 0.05 mM EGTA, 2 mM NTPs, and 0.4 U/µL RiboLock RNase Inhibitor (Thermo Fisher Scientific) (48) using 0.2 mg mL^−1^ of purified phi6 or phi13 NC. Purified phi6 protein P8 (0.36 mg mL^−1^) was added to the indicated reactions. Reactions were incubated at 30°C for 60 min and samples analyzed by electrophoresis in native agarose gel (TAE buffer, 0.5 µg mL^−1^ ethidium bromide). The gels were imaged (ChemiDoc Touch Imaging System, Bio-Rad) and the fluorescence of RNA molecules quantified using ImageJ. To calculate the transcription efficiencies of the phi6 and phi13 NC under different conditions, the total intensity of the ssRNA transcripts was divided by the total intensity of the genomic dsRNA.

### Cell lysis activity assay with phi6 P5

Phi6 protein P5 was acquired as side products from the purification of phi6 double-layered particles (59). The lytic activity of phi6 P5 was tested on chloroform-treated HB10Y cells as previously described (46, 76). P5-treated cell suspension was incubated at RT or 30°C for 2 h, and the optical density measured at 440 nm.

### Phi6 burst size at RT and 30°C

HB10Y cells were grown in liquid culture with aeration (200 rpm) first at 28°C to OD_550_ nm value ~0.45 and then at 23°C until the OD_550_ nm reached ~0.6 (≅4 × 10^8^ CFU mL^−1^). The cells were infected by phi6 at a multiplicity of infection of 10, divided into two cultures and grown at RT or 30°C. Ten minutes post-infection, a 1 mL sample was centrifuged (860 × *g*, 3 min) and the unabsorbed phage was determined from the supernatant and infective centers from the pellet. After an hour from the infection, the speed was lowered to 150 rpm and OD_550_ nm was followed. A 1 mL sample was taken after the cell lysis, centrifuged (860 × *g*, 3 min) and the phi6 titer of the culture was determined from the supernatant.

### Statistical analysis

Parametric (unpaired *t*-test with Welch’s correction) and non-parametric tests (Mann-Whitney/Kruskal-Wallis) were used to determine statistical significance. The data were computed using GraphPad Prism version 9.4.1 software. *P* values < 0.05 were considered significant.

## Data Availability

The data reported in this study are available upon request from the corresponding authors.

## SUPPLEMENTAL MATERIAL

Supplemental material spectrum.04029-25-s0001.pdf Fig. S1 to S11; Tables S1 and S2.

ASM does not own the copyrights to Supplemental Material that may be linked to, or accessed through, an article. The authors have granted ASM a non-exclusive, world-wide license to publish the Supplemental Material files. Please contact the corresponding author directly for reuse.
