# Supplementary material for: Temperature-modulated persistent dsRNA phage infection selectively excludes superinfection and promotes generation of new viral variants
Source: Microbiol Spectr. 2026 May 20;14(7):e04029-25. doi: 10.1128/spectrum.04029-25 (PMC13340061; doi:10.1128/spectrum.04029-25)
Supplement: Supplemental material — Fig. S1 to S11; Tables S1 and S2. [file spectrum.04029-25-s0001.pdf]

Supporting information

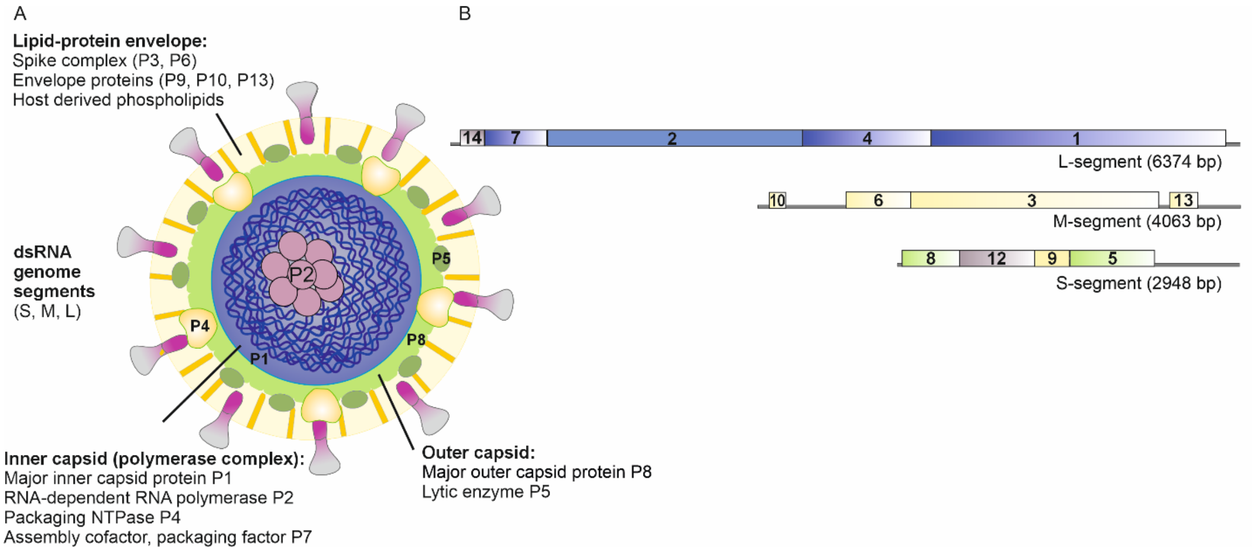

Figure S1. Schematic representation of bacteriophage phi6 virion (A) and genome (B). (A) The virion of phi6 contains three structural layers. Lipid-protein envelope (yellow) surrounds the nucleocapsid particle composed of two concentric protein shells: the inner capsid (polymerase complex; purple) and the outer capsid (green). The dsRNA genome is tightly packed inside the polymerase complex together with several copies of the viral polymerase subunit P2. (B) The genome of phi6 contains three dsRNA segments: L, M and S. Genes encoding components of the polymerase complex (purple) are clustered in the L-segment. Genes encoding envelope-associated proteins (yellow), the nucleocapsid shell (green) and viral non-structural proteins (gray) are in the M- and S-segments.

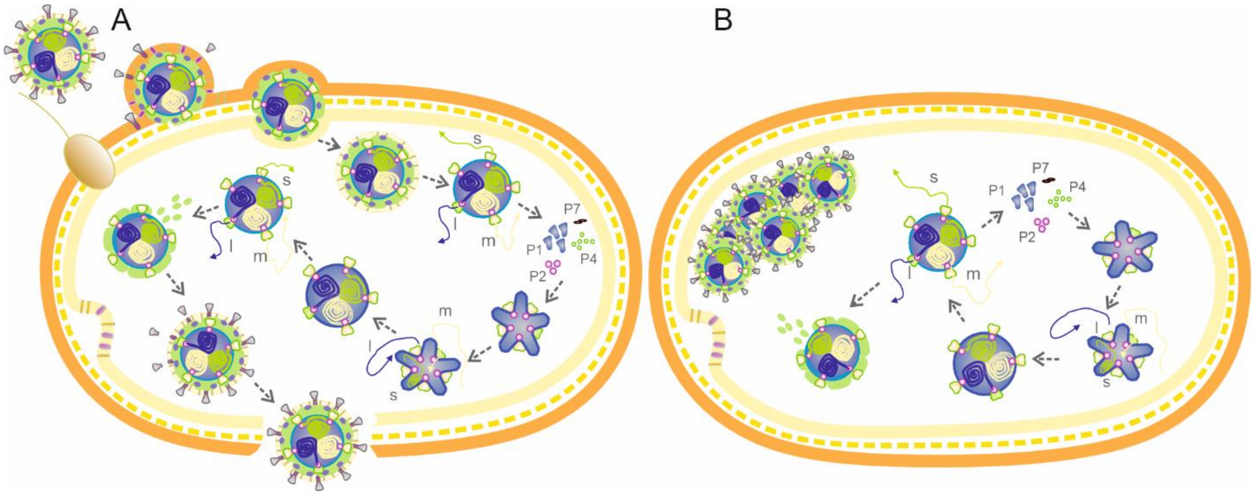

Figure S2. Schematic representation of phi6 lytic (A) and carrier cell infection (B) strategies. (A) The lytic life cycle of phi6 begins with the adsorption of the virion to type IV pilus of the host bacterial cell. As the pilus retracts, the virion is brought into contact with the bacterial cell surface. The viral envelope fuses with the host outer membrane, releasing the nucleocapsid to periplasmic space. The peptidoglycan layer is locally digested by the virion-associated lytic enzyme P5, after which the nucleocapsid (NC) enters the cytoplasm via an endocytic-like process. Disassembly of the NC surface shell made of protein P8 reveals transcriptionally active polymerase complex. P2 polymerase subunits, located inside the polymerase complex, catalyze the synthesis of phage mRNAs using the encapsidated genomic dsRNA molecules, S, M and L, as templates. The S-, M- and L-specific mRNAs (s, m, and l) are delivered into the cytoplasm, where the L-segment specific ssRNA molecules direct the synthesis of the proteins P1, P2, P4 and P7 which self-assemble into empty polymerase complexes. Phage-specific ssRNA molecules are packaged into the empty polymerase complexes in the order of S, M and L. After completion of the ssRNA packaging, the polymerase subunits synthetize a complementary strand for each encapsidated ssRNA segment. Subsequently, produced dsRNA molecules are transcribed by the polymerase into phage-specific mRNA molecules. In the host cytoplasm, the mRNA molecules direct the synthesis of the late phi6 proteins. The nucleocapsid surface shell assembles around the genome containing polymerase complex. Finally, the virion acquires the envelope from the host plasma membrane and spikes attach onto the virion surface. Mature phi6 virions are released upon host cell lysis, mediated by the viral lytic enzyme P5. (B) In carrier cell infection, the viral dsRNA genome is continuously expressed and replicated, and new viral particles are formed without cell lysis.

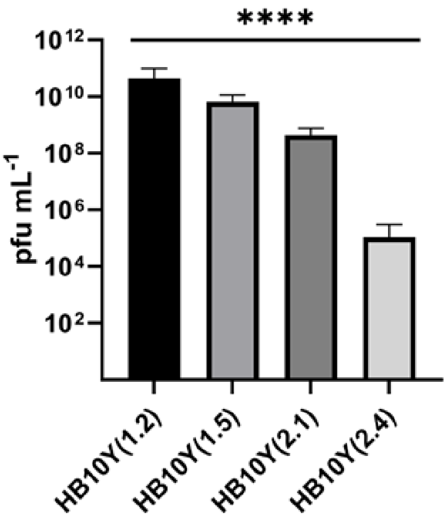

Figure S3. Release of infectious virions by cultures of spontaneous phi6 carrier cell lines. Number of phi6 phages (pfu mL^{-1}) liberated by spontaneous phi6 carrier cell lines HB10Y(1.2), HB10Y(1.5), HB10Y(2.1) and HB10Y(2.4) during overnight (18–19 h) cultivation in LB broth at room temperature. Values represent the mean + standard deviation (SD) of at least three biological replicates and are plotted on a logarithmic scale. ****P < 0.0001 (Kruskal-Wallis test).

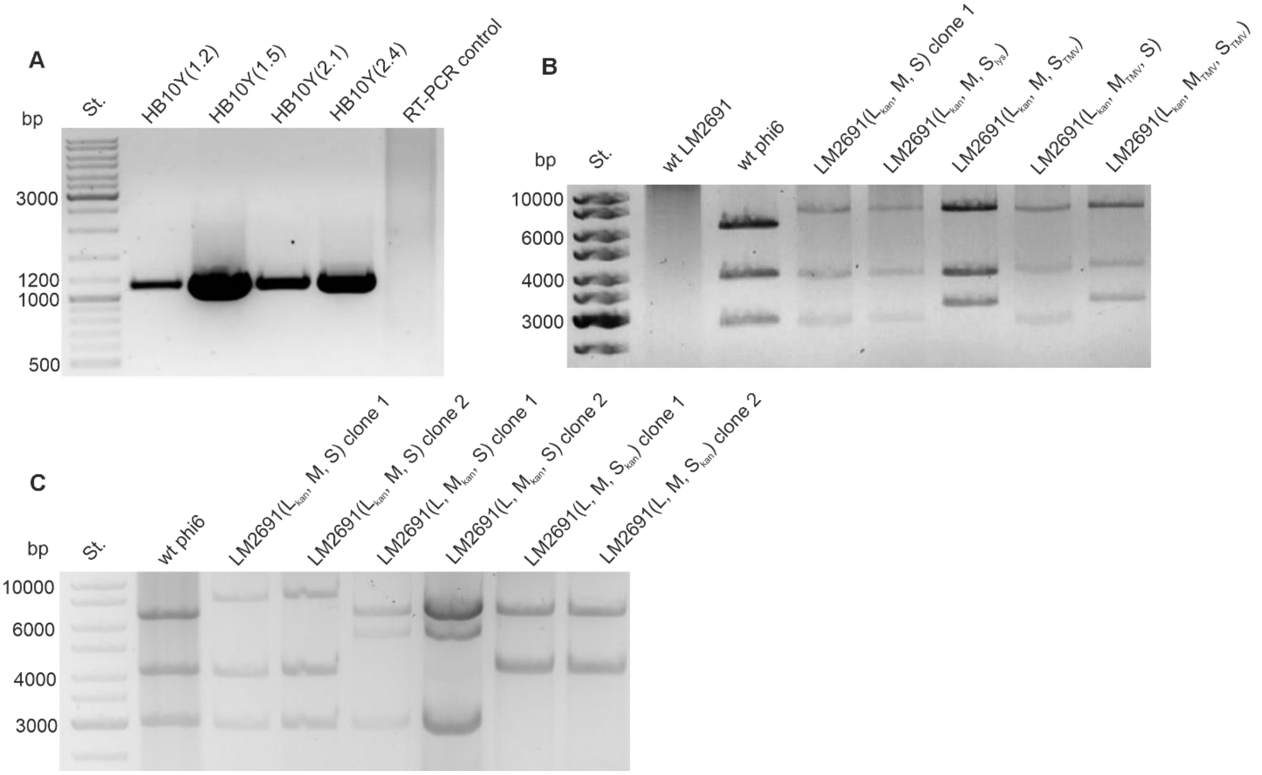

Figure S4. Production of phi6 carrier cells. (A) phi6-specific sequence was amplified from the spontaneous phi6 carrier cell lines by RT-PCR using primers specific for the phi6 gene *3* (Table S1), after which the amplification products (expected size 1141 bp) were analyzed by agarose gel electrophoresis. RT-PCR control is a PCR reaction without template cDNA. (B and C) Agarose gel electrophoresis analysis of total nucleic acid extracted from synthetic phi6 carrier cell lines. The extracted total nucleic acid from wild-type (wt) *P. syringae* LM2691 and genomic dsRNA of wt phi6 were used as controls. For LM2691(L, M, S_{kan}) strains, the separation of M and S_{kan} segments is not visible because of the small (46 bp) size difference of these two dsRNA molecules. For the expected sizes of the dsRNA molecules, see Table 1. GeneRuler DNA Ladder Mix (Thermo Scientific, 0.5 µg) was used as the size standard (st.).

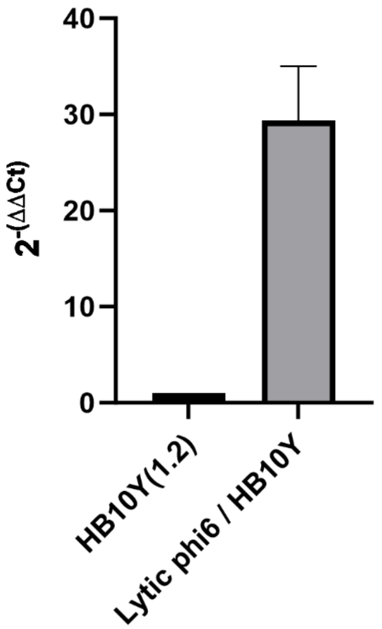

Figure S5. Virus load within *P. syringae* cells during lytic infection and carrier cell interaction. Quantification of phage phi6 gene *1* (encoding the major inner capsid protein P1) expression in HB10Y(1.2) carrier cells and HB10Y cells productively infected by phi6 (1 h post infection; lytic phi6) by RT-qPCR. RNA was extracted from both the infected and the carrier cell culture when the optical density at a wavelength of 550 reached 0.8. The mean mRNA expression levels, normalized to the *ftsZ* housekeeping gene of the host, are shown as relative fold change compared to strain HB10Y(1.2). HB10Y(1.2) has the relative quantification ($2^{-\Delta\Delta Ct}$) value of 1. Values represent the mean + SD of two independent biological replicates.

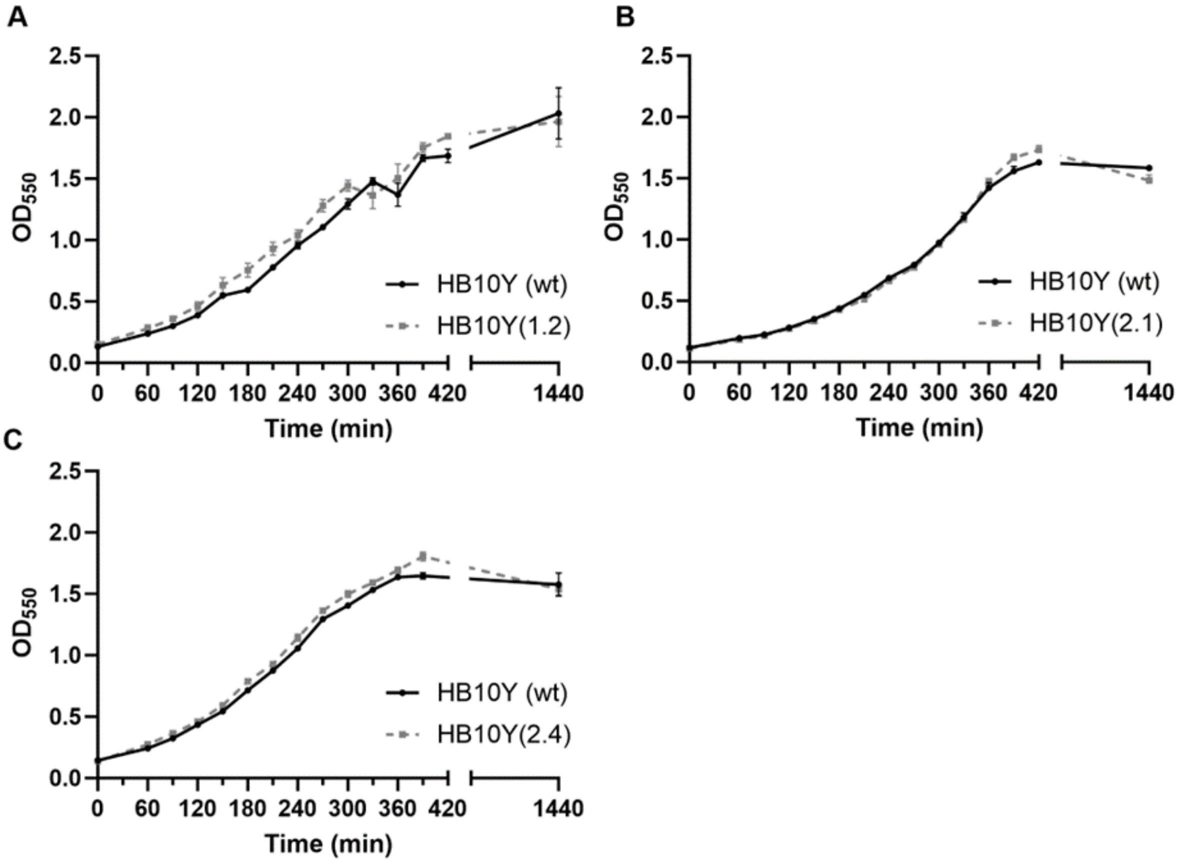

Figure S6. Impact of persistent phi6 infection on *Pseudomonas syringae* growth in LB medium at 28°C. Growth curves of spontaneous carrier cell lines (A) HB10Y(1.2), (B) HB10Y(2.1), and (C) HB10Y(2.4) are compared to wild-type (wt) HB10Y. Mean optical density at a wavelength of 550 nm (OD_{550}) ± standard error of the mean (SEM) of at least three replicates is presented.

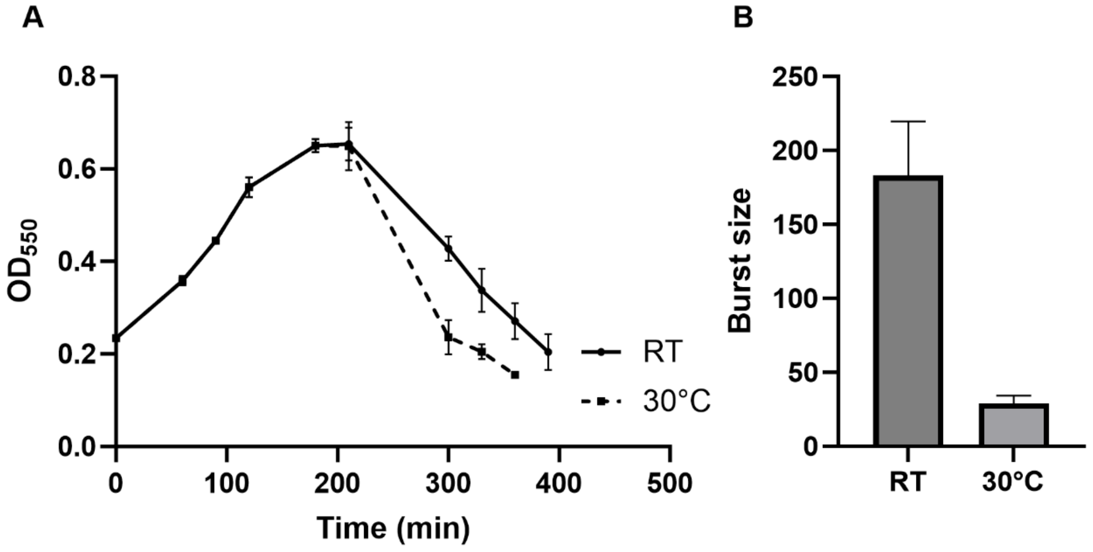

Figure S7. Burst size of lytic phi6 infection at different temperatures. (A) The lytic one-step growth curve of phi6 at RT and at 30°C. The HB10Y cells were grown first at 28°C to OD_{550} of ~ 0.45 and then at 23°C until the OD_{550} reached ~ 0.6 (= ~ 4 x 10^8 CFU mL^{-1}) at which point the cells were infected by phi6 at a multiplicity of infection of 10. The infected cell culture was divided into two separate cultures, which were then incubated at RT or 30°C. The OD values were followed until the cells had lysed. Mean OD_{550} value ± SEM of three independent biological replicates is presented. (B) The burst size of phi6 at RT and at 30°C. Values represent the mean + SD of three biological replicates.

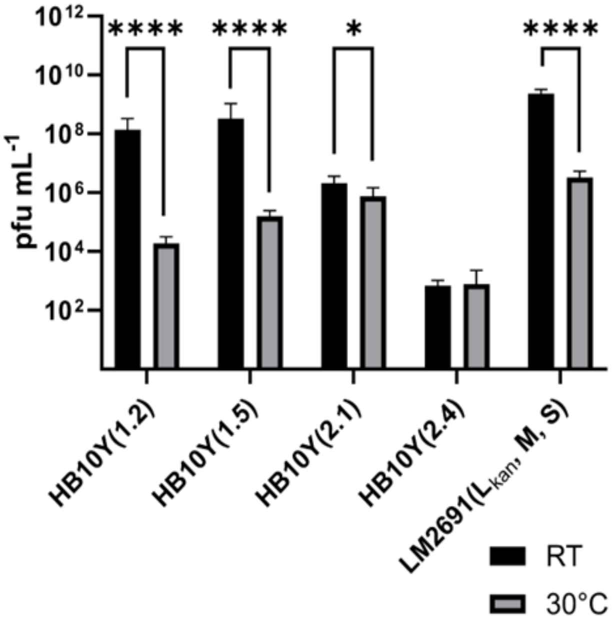

Figure S8. Effect of temperature on the stability of phi6 carrier cell interaction with its *Pseudomonas* host in low nutrient medium. Number of phi6 phages (pfu mL^{-1}) liberated by phi6 carrier cell lines during 24-hour cultivation in M9 broth at RT (black bars) and 30°C (gray bars). Values represent the mean + SD of at least three independent biological replicates, plotted on a logarithmic scale. **** $P < 0.0001$; * $P < 0.05$ (Mann Whitney test).

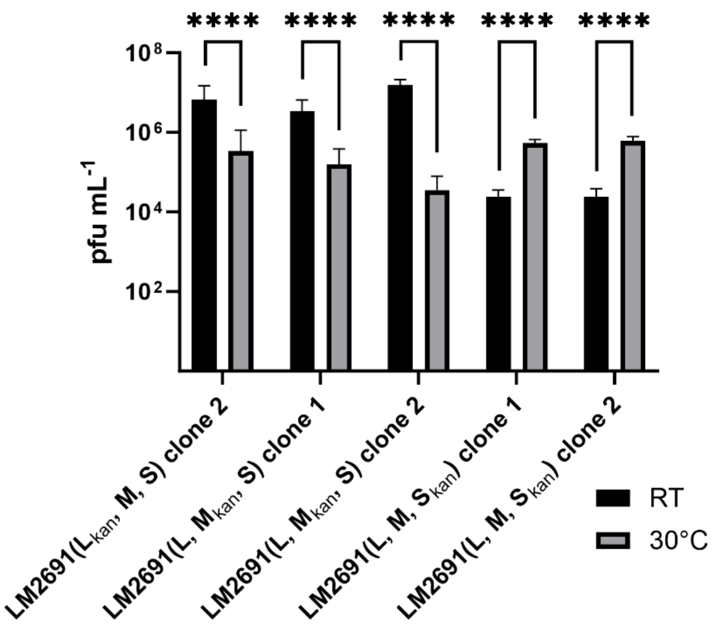

Figure S9. Effect of temperature on the stability of phi6 carrier cell interaction with its *Pseudomonas* host. Number of phi6 phages (pfu mL^{-1}) liberated by phi6 carrier cell strains during 24-hour cultivation in LB broth at RT (black bars) and 30°C (gray bars). Values represent the mean + SD of at least three independent biological replicates, plotted on a logarithmic scale. **** $P < 0.0001$ (Mann-Whitney test).

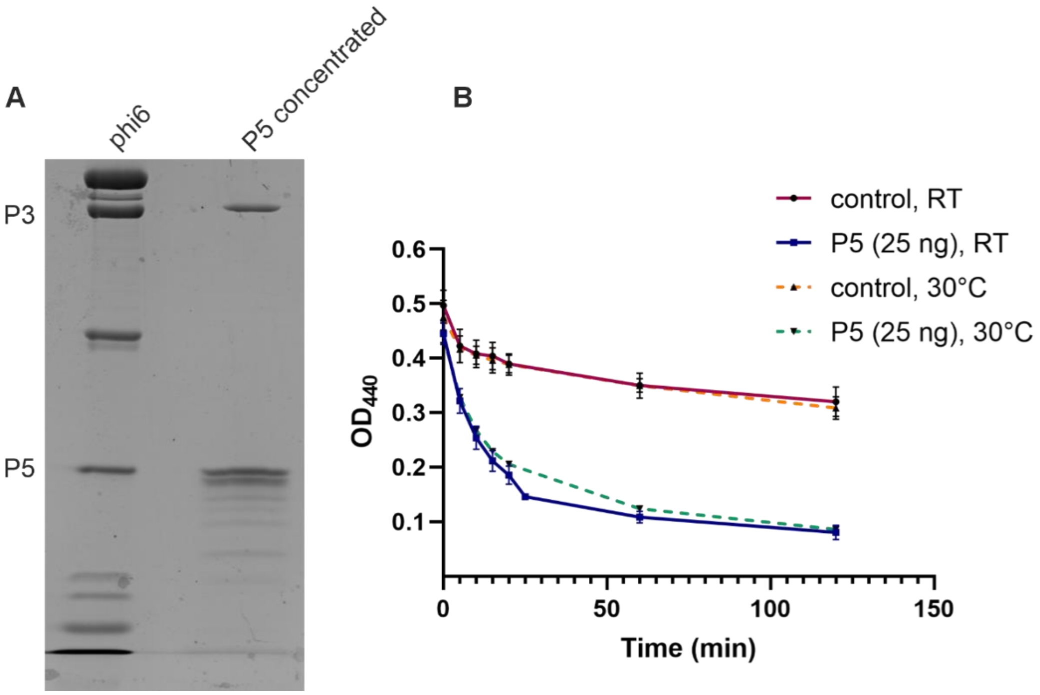

Figure S10. Temperature dependence of phi6 lytic enzyme P5. (A) Enrichment of protein P5. P5 was enriched using Amicon Ultra 50 000 NMWL centrifugal filter unit (Merck) collecting the flow-through and concentrated with 10 000 NMWL centrifugal filter unit (3220 × g, 4°C, Eppendorf 5810R, rotor A-4-62). The protein concentration was measured by Bradford assay. SDS polyacrylamide gel electrophoresis (15% acrylamide) of the enriched P5 fraction. The SDS-PAGE gel was stained with Coomassie brilliant blue and imaged (ChemiDoc™ Touch Imaging System, Bio-Rad). Purified phi6 virion sample was used as a control. The mobility of proteins P3 and P5 (24 kDa) are marked. (B) Effect of temperature on the lytic activity of phi6 P5 protein. P5 was added to chloroform-treated *Pseudomonas syringae* cells to attain a final protein concentration of 25 ng mL^{-1}, after which the cell suspension was incubated at RT or at 30°C for two hours. Turbidity of the cell suspension was measured as absorbance at 440 nm at specific time points. Chloroform-treated cells incubated without P5 addition at RT or at 30°C were used as a control. OD_{440} mean ± SEM of 2–4 independent biological replicates is presented.

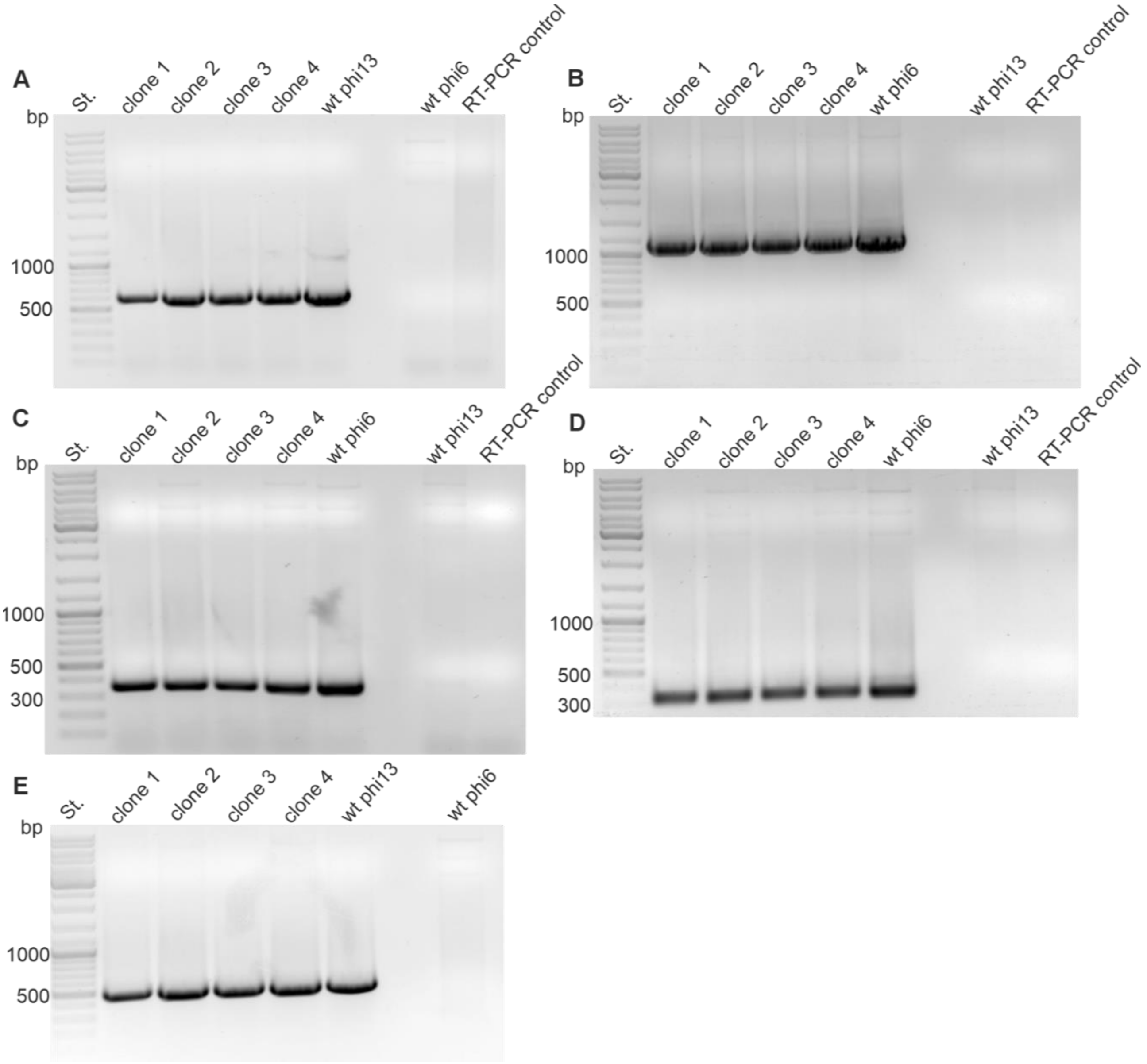

Figure S11. Characterization of progeny phages produced by phi6 carrier cells infected by phi13. Phi13 and phi6 specific sequences were amplified from the progeny phages by RT-PCR and the amplification products were analyzed by agarose gel electrophoresis. The extracted dsRNA from wild-type (wt) phi6 and phi13 were used as controls. GeneRuler DNA Ladder Mix (Thermo Scientific, 0.5 µg) was used as the size standard (st.). The RT-PCR products amplified using (A) phi13 S-segment specific primers (phi13 gene *5*; expected size 619 bp), (B) phi6 M-segment specific primers (phi6 gene *3*; expected size 1141 bp), (C) phi6 M-segment specific primers (phi6 gene *6*; expected size 368 bp), (D) phi6 M-segment specific primers (phi6 gene *10*; expected size 324 bp) and (E) phi13 L-segment specific primers (phi13 gene *1*; expected size 491 bp) are presented.

Table S1. Primers used in RT-qPCR and RT-PCR.

Primer target	product size (bp)	forward	reverse
housekeeping gene *ftsZ*[a]	108	AAGCACCTTCGGTCAACTAC	CCAGATCATCGTTAGGGTTCAT
phi6 L-segment gene *1*[a]	96	CAAGGAGATCACCGCTTTCA	GCCGACATACGATCAGAGATAC
phi6 M-segment gene *3*	1141	CTGAGGAAACGGCTCAACTG	GTTCCATTGATCATCGGCCC
phi6 M-segment gene *6*	368	GTGGTCGCCACCCTTAAGAA	ACACCGCCGATAATCGTACC
phi6 M-segment gene *10*	324	CAGGTGGACACCTCCTCAAC	ACCACGACGGAGAAAGAACC
phi13 L-segment gene *1*	491	GGCACTCCTCTCACGTATCG	GAGATACGCTCCACAGCCTC
phi13 S-segment gene *5*	619	ACGGTTATCGTCAATGGCGT	CCTTGAGTGCGTTTGACTGC

[a] Used for RT-qPCR

Table S2. The effect of temperature on phage phi6 infectivity.

**Incubation temperature**	**Phage phi6 infectivity** (pfu mL^{-1}) [a]
**4°C**	$(6.7 \pm 1.0) \times 10^{11}$
**RT**	$(6.0 \pm 1.5) \times 10^{11}$
**30°C**	$(4.1 \pm 0.8) \times 10^{11}$

[a] Phi6 suspension of approximately 3×10^{11} plaque forming units (pfu) was subjected to 24-hour incubation at 4°C, RT, and 30°C, after which the infectivity (pfu mL^{-1}) of phi6 was determined by plaque assay. The values represent the mean ± SD of three independent experiments.
